# Data-Driven Non-Linear Current Controller Based on Deep Symbolic Regression for SPMSM

**DOI:** 10.3390/s22218240

**Published:** 2022-10-27

**Authors:** Muhammad Usama, In-Young Lee

**Affiliations:** 1 Department of Electrical Engineering, Chosun University, 309, Pilmun-daero, Dong-gu, Gwangju 61452, Korea; 2 Department of Electronic Engineering, Chosun University, 309, Pilmun-daero, Dong-gu, Gwangju 61452, Korea

**Keywords:** deep learning, deep symbolic optimization, closed-loop control, data fitting expression, symbolic regression, metaheuristic algorithm

## Abstract

This study designs a simple current controller employing deep symbolic regression (DSR) in a surface-mounted permanent magnet synchronous machine (SPMSM). A novel DSR-based optimal current control scheme is proposed, which after proper training and fitting, generates an analytical dynamic numerical expression that characterizes the data. This creates an understandable model and has the potential to estimate data that have not been seen before. The goal of this study was to overcome the traditional linear proportional–integral (PI) current controller because the performance of the PI is highly dependent on the system model. Moreover, the outer speed control loop gains are tuned using the cuckoo search algorithm, which yields optimal gain values. To demonstrate the efficacy of the proposed design, we apply the control design to different test cases, that is varied speed and load conditions, as well as sinusoidal speed reference, and compare the results with those of a traditional vector control design. Compared with traditional control approaches, we deduce that the DSR-based control design could be extrapolated far beyond the training dataset, laying the foundation for the use of deep learning techniques in power conversion applications.

## 1. Introduction

Permanent magnet synchronous motors (PMSMs) have replaced DC and induction motors in various industrial applications such as electric cars, washers, numerically controlled machine tools, air conditioners, and robotics over the past two decades. PMSMs offer various advantages including a simple structure, high efficiency, higher power densities, low inertia, high torque-to-current ratios, and zero copper loss in the rotor winding [1,2]. Despite its benefits, the control of a PMSM to achieve high transient stability under all operating conditions remains difficult. PMSMs are multivariable, non-linear, and strongly coupled systems, which makes them particularly sensitive to parametric variations and disturbances, making it challenging to achieve a good dynamic control performance.

Traditional proportional–integral (PI) control strategies are primarily used to control PMSMs. However, the excellent performance of the system cannot be guaranteed because of non-linearities in the dynamic model equations caused by the non-linear features of the magnets and cross-coupling between the state variables [3,4]. Consequently, numerous control approaches have been proposed in recent years including predictive current control, artificial neural network (ANN) control, direct torque control, robust hysteresis current control, and H∞ control [5,6,7,8,9,10,11]. The predictive current control method in [5] predicts the current of the next sample and shows the fast convergence of the reference current to the actual motor phase current. However, the performance degrades with the parameter uncertainties [6]. The ANN-based control in [7,8] shows good performance. However, it is difficult to relate it to the network, making its application in scientific exploration challenging. In [9], the designed neural network (NN) exhibited good performance. However, there is a limitation to employing such networks because neural network architectures are typically difficult to understand, similar to a black box, difficult to interpret, and highly dependent on the information of the gradient search. Moreover, tuning the hyperparameters and weights is of primary concern because the accuracy of the network depends on this. Thus, an interpretable model that can derive meaningful information from complex datasets and extrapolate it outside the training dataset is required for machine learning for wide application in science. Purely data-driven techniques can perfectly fit the system dynamics. However, they only provide a black box model with low interpretability, rather than analytical numeric expressions. Direct torque and hysteresis controls are simple to implement. However, they have drawbacks, such as a variable switching frequency, large torque ripple, and high sampling rates for digital implementation [9,10]. However, H∞ control has a better convergence rate and effectively rejects load disturbances, although it is challenging to apply this to drive systems [11].

The modeling approach describes a model based on information obtained from the input and output data of a real system. It is important to note that the accuracy of a discovered model is highly dependent on the algorithms used to identify it and the data collected. Various identification strategies such as neural networks, fuzzy logic, reinforcement learning, and local linear regression (LR) have been proposed in the literature. Local linearization (local linear regression), complex calculations, and low explainability (NN) are common disadvantages of these approaches [12,13,14,15]. Artificial intelligence (AI) faces a major issue in determining the underlying mathematical equations that describe a dataset. This is a symbolic regression issue despite recent breakthroughs in training neural networks to solve complicated tasks such as ANN-based current control, ANN-based motor parameter identification, ANN torque observers, and ANN-based sensorless speed control [16,17,18]. However, symbolic regression approaches based on deep learning have been underexplored, particularly in power conversion applications. To the best of the authors’ knowledge, deep symbolic regression has not yet been used to design numerical models or model-based control laws. Therefore, as the state-of-the-art, deep symbolic optimization is employed to construct the proposed optimal current control design based on the analytical model. Symbolic regression based on deep reinforcement learning was used to construct an analytical model used for control design [19].

This study proposes an optimal current control design for the robust operation of a motor drive under standstill and transient operating conditions. A deep symbolic optimization technique was evaluated for the current control mechanism. To construct an analytical model that will replace conventional PI-based current control models, symbolic regression aims to obtain the following numerical expression that best fits the dataset. Deep learning combined with symbolic regression is employed. The goal is to construct a small mathematical expression model using a large model, that is an NN. This architecture exploits the representational capacity of a neural network to construct easy-to-interpret expressions while nullifying the requirement of interpreting the network. The resulting mathematical equation is easily readable. Moreover, an evolutionary algorithm, such as the cuckoo search algorithm, is employed for gain tuning the speed loop control. The unique contributions of this study are as follows:

(a) DSR-based optimal current controller;
(b) Training of the DSR-based controller on a recurrent neural network (RNN) in python;
(c) Utilization of an evolutionary algorithm for parameter tuning;
(d) Detailed study and performance analysis with a conventional control approach.

The remainder of this paper is organized as follows. The mathematical model of the SPMSM in a synchronous reference frame is presented in Section 2. Field-oriented control of the SPMSM using the conventional and proposed methods is described. The data generation and model training strategies are discussed in Section 3. The test results are presented in Section 4 under different operating conditions to demonstrate the fitting of the data and generate an analytical expression model. Finally, Section 5 presents the conclusions of this study.

## 2. Mathematical Model of SPMSM

The stator and rotor of the PMSM are both connected by an air gap magnetic field, which results in complicated electromagnetic interactions. The following assumptions were used to simplify the analysis without impacting the control performance: (a) The saturation of iron in the stator of the motor was neglected. (b) The effects of eddy currents and hysteresis were not considered.

Continuous-time electrical and mechanical SPMSM models in the synchronous dq reference frame were employed in this study and are defined as follows [20].

(1) $$\begin{array}{cc} L_{s}\frac{di_{d}}{dt} & =v_{d}-r_{s}i_{d}+\omega_{e}L_{s}i_{q}, \end{array}$$

(2) $$\begin{array}{cc} L_{s}\frac{di_{q}}{dt} & =v_{q}-r_{s}i_{q}-\omega_{e}L_{s}i_{d}-\omega_{e}\lambda_{m}, \end{array}$$

and the electromagnetic torque production becomes

(3) $$T_{e}=\frac{3}{2}\frac{P}{2}\left[\lambda_{m}i_{q}\right].$$

The mechanical torque equation in the non-linear form is expressed as follows:

(4) $$T_{L}=T_{e}-B\omega_{m}-J\frac{\omega_{m}}{dt},$$

where $i_{d}$ and $i_{q}$ are the armature currents; $v_{d}$ and $v_{q}$ are the stator voltages; $L_{s}$ denotes the stator inductance; $r_{s}$ denotes the stator resistance; $\omega_{e}$ is the electrical speed of the rotor. $\omega_{e}L_{s}i_{q}$ in (1) and $\left(-\omega_{e}L_{s}i_{d}-\omega_{e}\lambda_{m}\right)$ in (2) are dynamic coupling terms between the two-phase voltages $v_{d}$ and $v_{q}$. $\omega_{e}=P\omega_{m}$, where $\omega_{m}$ and *P* denote the rotor mechanical speed and pole pairs, respectively. $\lambda_{m}$ denotes the flux linkage of the magnet. $T_{e}$ and $T_{L}$ denote the electromagnetic and load torques, respectively. *B* and *J* are the viscous coefficient and rotor moment of inertia, respectively.

## 3. Field-Oriented Control of SPMSM

**Figure 1:**
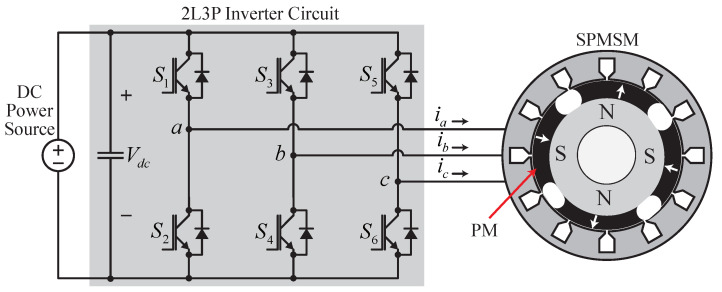
SPMSM powered by the 2L3P PWM inverter.

The field-oriented control of an SPMSM comprises a cascaded control structure with two individual PI controllers: one for the outer speed control loop and the second for the inner current control loop. Employing vector control technology makes it possible to achieve a robust dynamic performance of the SPMSM drive powered by a two-level three-phase (2L3P) PWM inverter, as shown in Figure 1. This section describes the conventional and proposed control schemes in detail. The results were used for the comparative analysis.

### 3.1. PI Speed Control Loop

In the application of electric motor drives, optimization-based control techniques are a well-established topic of study. In this study, cuckoo, a search optimization algorithm, was employed to tune the speed loop control gains, and tuned gains were used throughout. The original cuckoo search (CS) algorithm, in [21], was relatively easy to tune and provided only a small number of hyper-parameters. In this study, we slightly modified the algorithm. The basic form of the algorithm that we used is shown in Algorithm 1. It should be noted that we avoided the use of any metaphoric terms from the original algorithm’s literature such as *nests* or *eggs*. The cost function was calculated as the integral time absolute error (ITAE). We selected the ITAE as the error performance index because it focuses on more accurate steady-state error-free control. Hence, we expected good overall optimization of the speed control loop. It is calculated over a time interval as given in Equation (5). At each data point, we took the residual difference between the reference and actual speeds as:

(5) $$\mathrm{ITAE}=\int_{0}^{\infty }t\left|\omega_{i}^{ref}-\omega_{i}^{act}\right|dt.$$

In Equation (5), $\omega_{i}^{ref}$ is the reference speed and $\omega_{i}^{act}$ is the achieved speed at the *i*th time instant.

**Algorithm 1** Cuckoo search algorithm**Input:** (*n*, *p*, *k_max_*)
**Output:**
*x*_0_
 1: Randomly initialize *n* candidates ${\bar{x}}_{i}=\left(i=1,2,...,n-1\right)$ 2: Calculate the error $f_{i}$ of each candidate $x_{i}$ 3: Sort the population in ascending order of error $f_{i}$ 4: **for** ($k=0:k<k_{max}$) **do** 5:    **for** (i=0:i<p*n) **do** 6:      Randomly pick a candidate $x_{i}$ with error $f_{i}$ 7:      Generate $x_{j}$ by mutating $x_{i}$ using Equation (6) 8:      Calculate error $e_{j}$ of $x_{j}$ 9:      **if** ($e_{j}<f_{i}$) **then**10:         Replace $x_{i}$ with $x_{j}$11:      **end if**12:    **end for**13:    Sort the population in ascending order of error $f_{i}$14:    Randomly initialize (p*n) worst candidates15: **end for**

The algorithm used to tune the gain parameters for the speed control loop is described as follows. In Lines 1–3, a population of *n* candidates is randomly initialized and arranged in ascending order of their error scores. Lines 4–14 describe the main *for* loop that operates for a defined number of iterations, $k_{max}$. Lines 5–11 perform a local search inspired by the behavior of cuckoo birds. A new candidate was generated by mutation and was selected randomly from the population using Equation (6).

(6) $$x_{i}\left(k+1\right)=x_{i}\left(k\right)+\alpha ⊕Lé\mathrm{vy}\left(\lambda \right),$$

where ⊕ denotes the elementwise multiplication. The random step length is drawn from a Lévy distribution as follows:

(7) $$Lé\mathrm{vy}∼u=t^{-\lambda },\;\left(1<\lambda \leq 3\right).$$

**Figure 2:**
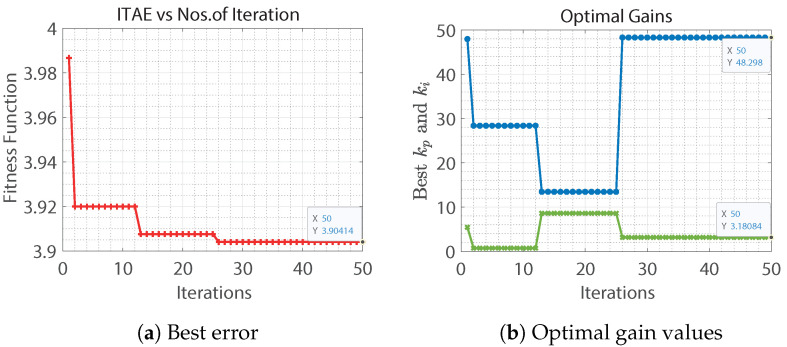
Cuckoo search algorithm for speed control loop.

The mutation is applied using the Lévy flight, which is a random walk, whereas the random step length is drawn from the Lévy distribution given by Equation (7). If the new candidate is more suitable, this replaces the previous candidate. Line 13 applies to the parasitic operator; (p*n) candidates with the worst error values were replaced by randomly generating candidates according to probability *p*. This operator helps maintain diversity in the population. The best error function and gain values were obtained from the aforementioned cuckoo search algorithm shown in Figure 2.

### 3.2. Conventional Current Control Scheme

The PI controller is a conventional control scheme, which is mostly employed in current control owing to its easy implementation. PI controllers are highly model-dependent and have parametric uncertainties. The performance of the control loop is degraded, which affects the closed-loop control performance [22]. The transfer function employed to tune the gain parameters was obtained without the inclusion of coupling terms, which caused decoupling inaccuracies [23]. The transfer function of the plant utilized to tune the gain parameters is as follows:

(8) $$T\left(s\right)=\frac{1}{sL_{s}+r_{s}},$$

where *s* denotes the Laplace variable. The closed-loop transfer function of the current control is derived as follows:

(9) $$\frac{i(s)}{i^{*}\left(s\right)}=\frac{K_{p}s+K_{i}}{s^{2}L_{s}+\left(r_{s}+K_{p}\right)s+K_{i}},$$

where *i* is the actual motor phase current and $i^{*}$ is the reference motor phase current. The output of the PI controller was a voltage command in the synchronous reference frame, which was further transformed into the abc reference frame using the well-known inverse Park transformation. Moreover, the controller aims to increase the accuracy by minimizing the stability error and provides fast convergence to the reference currents. However, the controller does not guarantee stability against load disturbances or input saturation limits.

### 3.3. Proposed Current Control Scheme

In artificial intelligence, identifying a tractable numerical expression that best explains a dataset has been a long-standing problem in the generation of new physical knowledge and insights; this challenge is known as symbolic regression [24]. Conventionally, symbolic regression is performed using genetic programming, where the population of numerical expressions is generated by employing metaheuristic algorithms to determine the best fit of the data. Experimental data are used to extract the underlying laws of physical systems using this method. However, genetic programming does not scale well with large systems owing to the combinatorial scope of the issue and is prone to overfitting [25,26].

**Figure 3:**
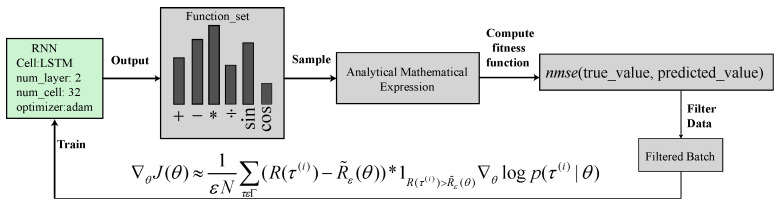
Deep symbolic regression core architecture.

Deep symbolic regression (DSR) is a deep learning method for symbolic regression based on the recently proposed policy gradients. DSR exhibits remarkable results in terms of recovering symbolic equations compared to genetic programming in various test cases [19]. Here, we present the application of DSR for the design of an optimal current controller. The algorithm takes advantage of the deep neural network and generates interpretable and generalized models. An overview of the core algorithm is shown in Figure 3. The training was performed offline using Python by employing the inverse normalized mean-squared error (INMSE) bounded by a sigmoid activation function. The risk-seeking policy gradient computes the empirical quantile of the reward and filters out data with less reward depending on the epsilon value. In the model structure, the space of the numerical equation was discrete. However, in the model parameters, the space is continuous. Provided the data (X,y), where at each point, $X_{j}\;\epsilon \;R^{n}$ and $y_{j}\;\epsilon \;R$, DSR seeks to determine the function $f\;:\;R^{n}\;\to \;R$ that best fits the data.

A recurrent neural network (RNN) in the DSR outputs a distribution of the numerical expressions. The expressions are chosen randomly from the distribution, instantiated, and assessed to fit the dataset. This fitness was utilized as the reward signal to train the RNN using a unique risk-seeking policy gradient technique, where the goal was to optimize the best-case performance of the policy for the expected reward, unlike the best average performance for the standard policy gradient. The RNN modifies the possibility of numeric expressions in relation to its reward as training progresses, assigning higher probabilities to better the numeric expressions. The reward function is computationally less expensive than other techniques; we fed the input data to the sequential numeric expression generated from the RNN. The predicted output values were compared with the true values with the fitness function INMSE as a reward signal, which takes only a few microseconds. The final expressions with a maximum reward 0.738 for the $v_{d}$ command reference and 0.7504 for the $v_{q}$ command reference were obtained as follows:

(10) $$\begin{array}{cc} v_{d}^{*} & =\left(-2x_{1}+x_{2}+x_{3}\right)(x1\left(-4x_{1}+4x_{2}+2x_{3}\right)+4x_{1}-3x_{2}-2x_{3},\\ v_{q}^{*} & =3x_{1}(x_{2}\left(-2x_{1}+2x_{2}+x_{4}\right)+7x_{2}-sin\left(x_{1}\right)+cos\left(x_{2}\right), \end{array}$$

where $x_{1}=e_{d}$, $x_{2}=e_{q}$, $x_{3}=\int e_{d}$, and $x_{4}=\int e_{q}$, respectively.

The control scheme of the proposed DSR-based current controller is described as follows:

(a) Measure the current errors and their integration in the synchronous reference frame at sampling time $T_{s}$.
(b) The integral error information is fed into the system, ensuring that there is no steady-state error in the reference tracking.
(c) The DSR algorithm employing the risk-seeking policy gradient generates numerical expressions that are easy to understand and fit the data.
(d) The generated expressions are employed in an online model as an optimal current controller.

The test results are compared with those of conventional methods in the next section to verify the effectiveness of the proposed control design.

## 4. Performance Analysis of Field-Oriented Control

The comprehensive analysis and comparison study of the proposed and conventional closed-loop vector control of a surface-mounted permanent magnet synchronous motor drive are presented in this section.

### 4.1. Test Setup

**Figure 4:**
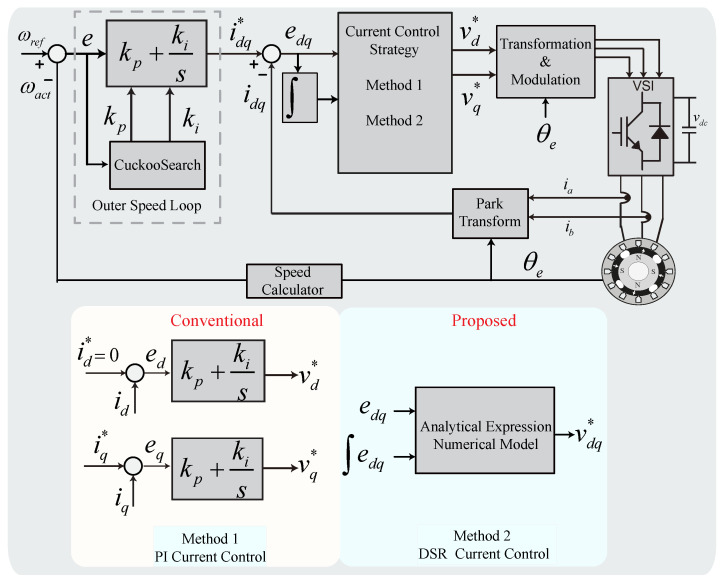
Conventional and proposed field-oriented control architecture of SPMSM.

To demonstrate the efficacy of the DSR-based optimal current control technique and compare its performance with that of the traditional current control, we used Python 3.6 and Matlab Simulink (2022a) to implement the models, as shown in Figure 4. We conducted training on the generated datasets. The training was performed offline, and the mathematical model generated from the DSR was employed online. The model was designed to acquire the reference voltage commands using the proposed and conventional schemes on a laptop with an Intel^®^ Core i7-1195G7 2.90 GHz CPU, 16 GB RAM, and NVIDIA GeForce^®^ RTX laptop GPU 3050, running Windows10 64 bits.

### 4.2. Training Procedure

The training process consisted of three main steps:

(a) Data generation and processing using Matlab.
(b) Setting up a deep symbolic regression algorithm in Python, tuning the hyper-parameters.
(c) Generation of analytical, numerical expressions that fit the dataset.

To train DSR, datasets were generated by performing extensive Matlab simulation tests using a linear controller as the baseline control scheme. Various reference signals consisting of step, sinusoidal, and sawtooth signals were fed to the SPMSM controlled by a linear current controller. The training dataset comprised 30 test scenarios. For each test condition, the simulation was run under numerous operating conditions such as the input reference signals, desired speed, load torque, and sampling time. The stored data with the input features and their targets were employed for supervised learning to solve the non-linear regression problems. The training was performed offline, employing both Matlab and Python to generate an analytical–numerical model that fits the dataset. After proper training, the generated analytical–numerical model was used by replacing the linear current controller. To verify the performance of the proposed current control method, we evaluated the dynamic performance of the SPMSM using the control parameters listed in Table 1, by employing conventional and proposed current controls under various operating conditions.

### 4.3. Test Results and Discussion

To demonstrate the efficacy of the proposed control design, different test cases were studied, and the results were compared with those of the traditional control design. The drive control parameters and optimization and deep learning hyperparameters are listed in Table 1 and Table 2. The gain parameters of the closed-loop vector control of the outer loop were tuned offline by an evolutionary algorithm and employed online in the closed-loop vector control. The data for the DSR training were generated by employing a linear current controller, and the generated analytical expression was utilized online, compared to a linear controller in the closed-loop control system. The test results of the proposed and conventional linear control were compared, showing that the proposed method converges to the data perfectly, as desired in PI control, but without the need for any tuning parameters and fitting dataset online. These generated expressions are easy to understand and can be utilized online in the control architecture. These expressions are non-linear in nature and show good compatibility with the dataset compared to the linear controller (PI). The test cases were as follows.

Case I 

**Figure 5:**
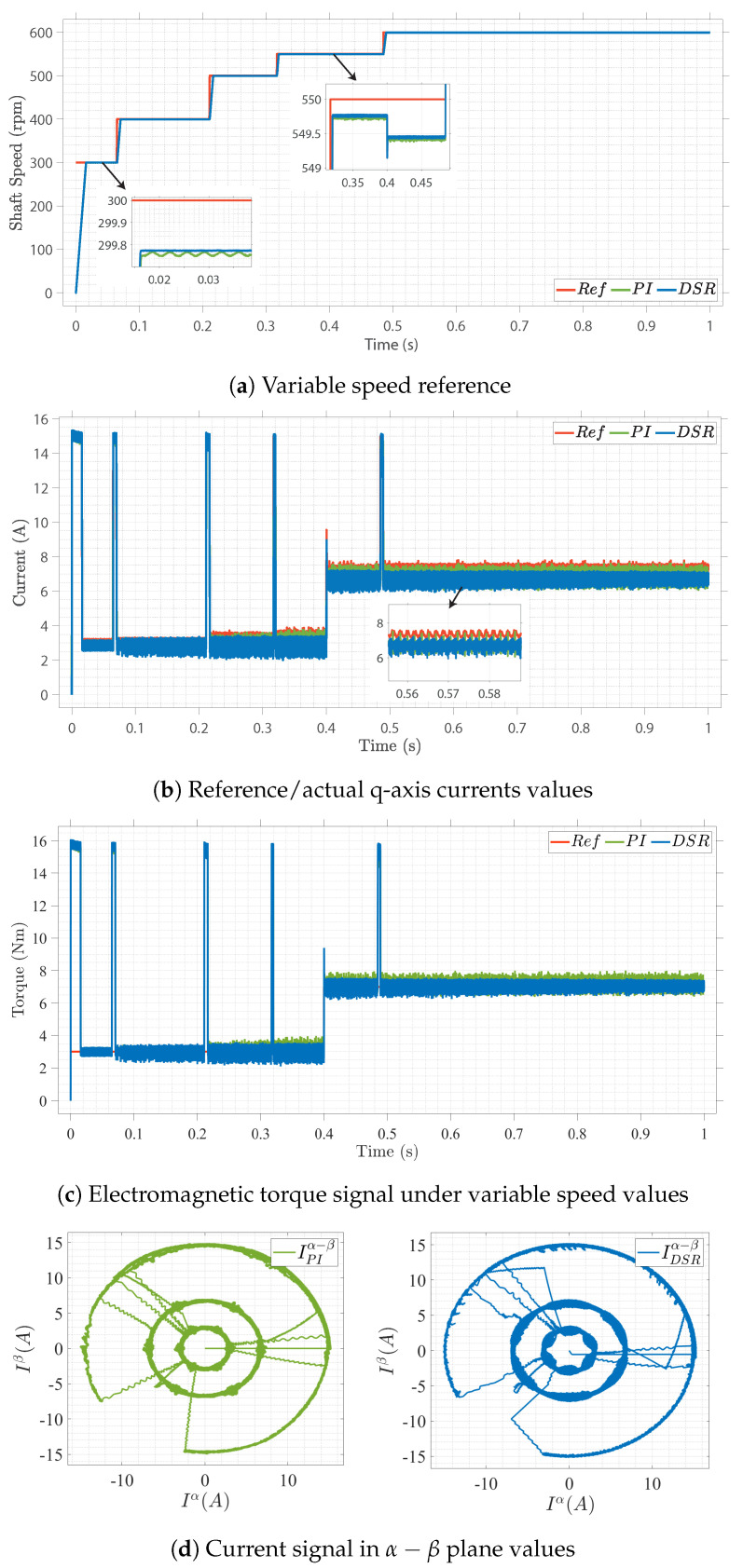
Closed-loop field-oriented control of SPMSM under speed variations.

The closed-loop control performance under a varied-speed input reference is shown in Figure 5. Figure 5a shows the speed convergence for a varied-speed reference input. The tuned gain parameters were used for the outer speed loop, which is responsible for generating the quadrature reference current $\left(i_{q}^{*}\right)$. The steady-state performance and speed response at increasing load torque at 0.4 s show that the proposed closed-loop design has less ripple and steady-state error than the conventional control design, as can be observed in Figure 5b,c. The results of the proposed design show excellent standalone and dynamic performance under various working conditions. Moreover, the generated numerical analytical model is well fit to online data, as shown in Figure 5b–d, where the controller converges to the reference values.

Case II

**Figure 6:**
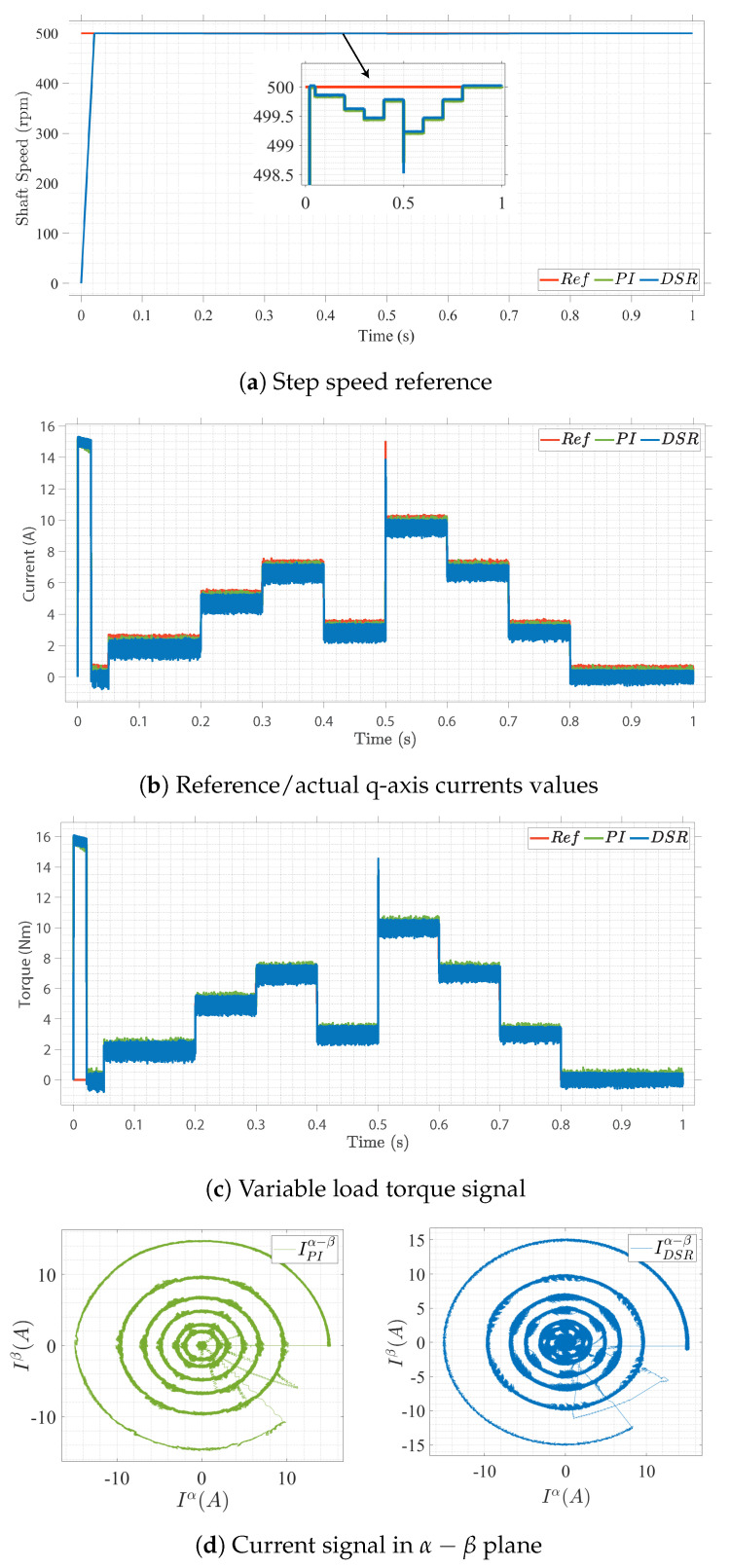
Closed-loop control performance under load variations.

The closed-loop control performance under varied-load references with a constant-speed input is presented in Figure 6. The load torque was gradually increased from 0 Nm to 7 Nm at 0.3 s. At 0.4 s, the load torque decreased to 3 Nm and stayed at 3 Nm for approximately 0.1 s, then increased to 10 Nm and, finally, gradually decreased to 0 Nm at 0.8 s. In Figure 6a, under load variation, the speed error of the proposed control design was lower than that of the conventional PI. Moreover, the current convergence to the reference values and the torque ripples were lower in the DSR-based current controller than in the PI-based current controller, as shown in Figure 6b–d. The proposed system provided better readings, proving its dynamic performance under different operating conditions.

Case III 

**Figure 7:**
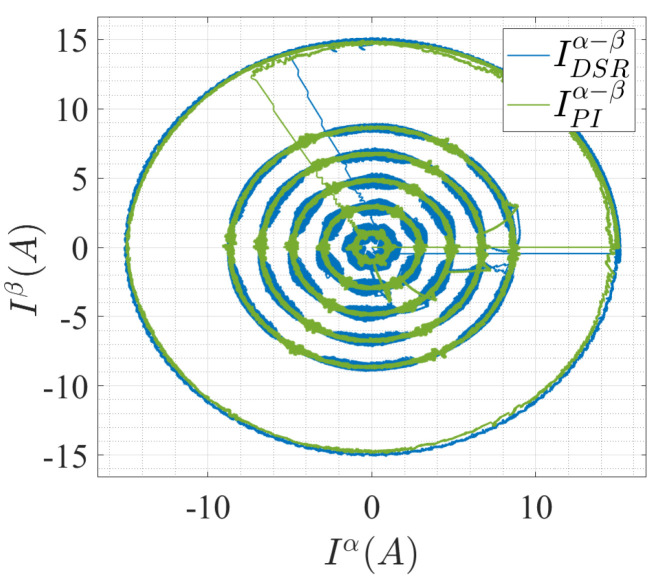
αβ stator current under variable load and speed reference.

Furthermore, in the third case, the parameters of the system were varied, keeping the speed constant. Figure 7 shows the α−β stator current planes for the two control strategies. The analytical model generated offline from the DSR fit the online data well and converged the actual current signal to the current reference values. This shows that the DSR-based current controller can replace the PI controller in which the performance is highly dependent on the control gains. By replacing the PI with a DSR-based current controller, the limitation of tuning the proportional–integral (PI) gains can be eliminated.

Case IV 

**Figure 8:**
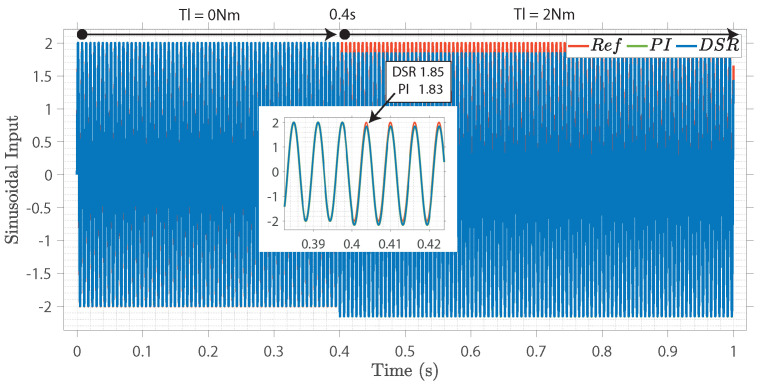
Closed-loop field-orientated control performance: sinusoidal step input.

Figure 8 shows the speed responses of the conventional and proposed closed-loop controls under a sinusoidal speed step reference. Initially, the load torque was 0 Nm. At 0.4 s, the load torque was increased to 2 Nm, and the speed convergence is shown. The proposed closed-loop controller had a high convergence rate compared to the conventional closed-loop control design. Moreover, the results imply that the analytical model converged efficiently to the desired input.

Case V

**Figure 9:**
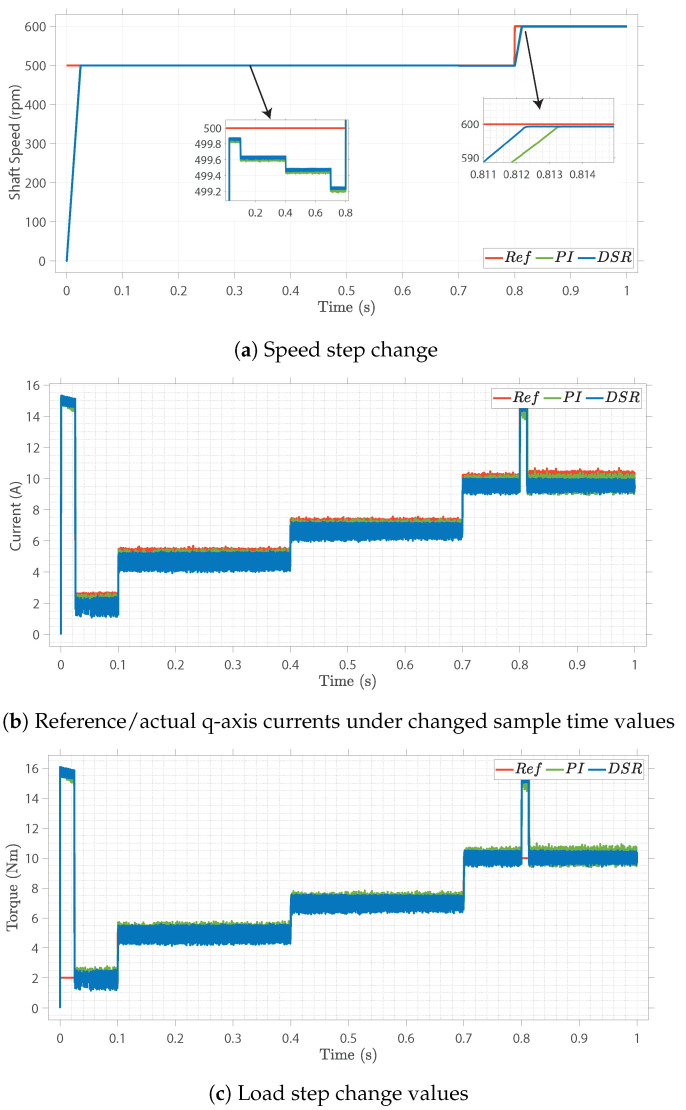
Load variation and sampling rate effect on closed−loop control.

Finally, the performance of the controller was analyzed based on the change in the sample time under load disturbance and speed variations, as shown in Figure 9. The sample time was selected as 20 μs, as it can be clearly observed that the response of the proposed design is better than that of the conventional design. Thus, the numeric expressions fit the dataset online and neglected the impact of changing the operating conditions. The transient response of these systems was considered, and the proposed design outperformed the conventional PI control strategy.

The test results verified the effectiveness and usefulness of a non-linear controller based on the DSR strategy. The quantitative performance analysis of both control designs is shown in Table 3. The generated models based on higher rewards were employed in this study, and the models could extrapolate beyond the training dataset and fit the desired reference data input online. The test results showed that the proposed algorithm had a similar tracking performance with minimal speed error and torque ripple compared to the linear controller.

## 5. Conclusions

A non-linear control design employing deep learning combined with symbolic regression was implemented in this study. The DSR-based non-linear controller eliminated the limitations of conventional linear control design, where the tuning gain parameters were highly significant. Optimal gain parameters play an important role in the dynamic and excellent performance of closed-loop linear control design. For the outer speed loop, the gain parameters were tuned by the cuckoo search algorithm, which provided optimal gains using fewer parameters than other metaheuristic schemes. The q-axis current was generated from the outer speed loop, whereas the voltage commands were generated from the DSR-based non-linear controller, whose main goal was to fit data that have never been seen and converge the actual currents to the reference values, providing good dynamic closed-loop control performance. The utilization of DSR for generating a numerical expression that provides voltage commands is the state-of-the-art in motor drive systems and paves the way for the application of deep learning techniques in power energy conversion applications. In the future, a deep symbolic optimization scheme will be studied for speed sensorless mechanisms, as well as the classification of gating signals for 2L3P VSI. Moreover, the DSR will be optimized by tuning the hyper-parameters of the RNN to achieve numerous better-generated expressions with higher rewards.

## Figures and Tables

**Table 1:** SPMSM parameter values.

Parameter	Description	Value
$T_{s}$	Sampling time	1 μs
$F_{pwm}$	Switching frequency	10 kHz
$r_{s}$	Stator resistance	0.2 Ω
$L_{s}$	Stator inductance	0.000835 H
$\lambda_{m}$	Flux linkage	0.175 Wb
*P*	Poles pairs	4
*J*	Inertia	0.0027 kg·m${}^{2}$
*B*	Damping coefficient	0.000049 Nsm${}^{-1}$

**Table 2:** Cuckoo optimization and RNN hyper-parameter values.

Parameter	Description	Value
$k_{max}$	Maximum iterations	50
*n*	Population size	15
*p*	Parasitic probability	0.25
kpω	Upper and lower bounds	[0, 50]
kiω	Upper and lower bounds	[0, 10]
*O*	Optimizer	Adam
*L*	Learning rate	0.001
*C*	Cell	LSTM
*B*	Batch size	500
*N*	samples	20,000
ϵ	epsilon	0.2

**Table 3:** Comparison analysis of linear and data-driven control scheme.

Feature	PI	DSR
Plant model	dependent	independent
Tuning	required	N/A
Control dynamics	good	good
Speed and torque ripples	high	low
Computational burden	low	low
